# Informal welders’ occupational safety and environmental health risks in northwestern Tanzania

**DOI:** 10.1371/journal.pgph.0002923

**Published:** 2024-02-28

**Authors:** Leah Magoha, Elias C. Nyanza, Moses Asori, Deborah S.K. Thomas

**Affiliations:** 1 Occupational Health and Safety Authority, Lake Zone Office, Mwanza, Tanzania; 2 Department of Environmental, Occupational Health, and GIS, School of Public Health, Catholic University of Health and Allied Sciences, Bugando, Mwanza, Tanzania; 3 Department of Geography & Earth Sciences, The University of North Carolina at Charlotte, Charlotte, NC, United States of America; Muhlenberg College, UNITED STATES

## Abstract

Often with minimal formal training and protections, informal welders face significant occupational health and safety (OSH) risks. This cross-sectional study of 219 adult informal welders at 70 informal welding sites in Mwanza City, Tanzania aimed to: 1) capture knowledge and awareness of occupational risks and safety precautions, training, and self-reported work-related injuries and illness and 2) observe worker use of personal protective equipment and site safety. We hypothesized that knowledge, awareness, and site inspections would improve use of PPE and that improved safety and site inspections would reduce self-reported injuries and illness. A generalized linear model (GLM) was used to model all relationships. Robust standard error estimation was used to avoid overestimation of parameters. Having a post-secondary education (aβ = 1.01, 95% CI: 0.962, 1.061; *p* = 0. 0679), having training in OSH (aβ = 0.927, 95% CI: 0.872, 0.984, *p* = 0.014), increased knowledge of occupational risks (aβ = 1.305, 95% CI: 1.143, 1.491; *p*<0.001), and knowledge of safety measures (aβ = 1.112, 95% CI: 0.881, 1.404; *p* = 0.372) increased PPE use by 1%, 7.3%, 30.5%, and 11.2% respectively. Workers who used PPE were less likely to experience fire explosions (AOR = 0.149, 95% CI: 0.029, 0.751; *p* = 0.02), radiation exposure (AOR = 0.097, 95% CI: 0.016, 0.579, *p* = 0.01) or electric shocks (AOR = 0.012; 95% CI: 0.001, 0.11, *p*<0.001). Having increased knowledge of safety practices also decreased the odds of fire explosions (AOR = 0.075, 95% CI: 0.018, 0.314; *p*<0.001). Those with higher knowledge of occupational risk (aβ = 1.57, 95% CI: 1.404, 1.756; *p*<0.001) and safety measures (aβ = 1.628, 95% CI: 1.34, 1.978; *p*<0.001) were more likely to have more positive attitudes towards safety practices. Our findings suggest that comprehensive targeted interventions including increased knowledge of occupational risks, safety practices, and occupational health law through training, along with enforcement and inspection by government officials, would benefit the environmental and occupational health for informal welders.

## 1. Introduction

In Africa, an estimated 71.9% of non-agriculture employment is informal. Often with minimal formal training and protections, workers face significant occupational health and safety risks with approximately 2 million people perishing every year from work-related causes globally [1, 2]. In Southern Africa, an estimated 18,000 people die from work-related illnesses every year and 13 million are injured and 67,000 contract work-related illnesses [3]. In recent years, Tanzania has experienced increased development, including large-and small-scale industries, building, and manufacturing, requiring welding. Most welders in Tanzania work in the informal sector with limited adoption of safety precautions [3–5].

The welding process joins and cuts metal using a flame or an electric arc and other heat sources to melt and cut or to melt and join two pieces of metal [6]. There are several types of arc welding, including stick or shielded metal arc welding (SMAW), the gas-shielded methods of metal inert gas (MIG), tungsten inert gas (TIG), plasma arc welding (PAW), and submerged arc welding (SAW) [7, 8]. The most common welding method used by informal welders is the SMAW, which uses electricity as a source of heat [9, 10]. Informal welding sites, like many other small-scale industries, are characterized by low capitalization, and minimum investment in safety precaution procedures, putting those involved and the communities around them at risk [6].

Welding is a hazardous profession with many health and safety risks to workers, including heat exposure, burns, dangerous flying objects, radiation exposure, noise pollution, fumes (for example particulate matter ‐ PM_2.5_ and PM_10_), electrocution, and poor ergo-dynamics [6, 11, 12]. These occupational hazards can result in temporary and/or permanent injury, short- or long-term adverse health effects, discomfort, and even death to workers [6, 11]. Short-term effects include cuts and burns, irritation of the eyes, nose, chest, and respiratory tract, muscle aches, coughing, fatigue, and nausea. Long-term health effects include an increased risk of pulmonary siderosis due to exposure to iron dust, lung, larynx, and potential cancers from agents found in smoke [6, 12]. Many of these hazards can be minimized with adherence to occupational health and safety guideline, using personal protective equipment (PPE), maintaining equipment, practicing electrical safety, and employing proper hazard control measures [6, 9–11, 13]. Welders should not weld or cut unless wearing the necessary PPE to protect all areas of their body from injury during welding or cutting [14]. Proper welding PPE consists of a welding hood with a face shield, a respirator or mask, safety glasses, earplugs, gloves, long-sleeved jacket, closed-toed shoes, and ear plugs [14].

The Tanzanian Occupational Health and Safety Authority (OSHA) regulates and oversees all occupational health and safety (OHS) across the Tanzania mainland, as stipulated by the *OHS Act Number 5 of 2003* and the *OHS Policy of 2009* [15, 16], aiming to promote and maintain physical, mental, and social well-being of workers across occupations [14]. However, the Tanzanian laws and policies are not as comprehensive as they could be to protect a broad population. Further, safety service provision and inspection from the government is scarce [3]. Typically, adherence to various OHS guidelines is primarily the responsibility of workers and their managers at workplaces. Yet, knowledge of, and adherence to, workplace safety practices and guidelines, remains a significant challenge [3]. As such, coupled with limited site inspection, safety practices informed by the laws and practices is often quite low among welders [17].

In parallel to rapid population growth across Tanzania from about 44.9 million in 2012 to 61.7 million in 2022 [18], Mwanza City, the third largest city in Tanzania and the largest in the Lake Zone along Lake Victoria, has also increased. This substantial population growth has spurred building and development with a significant increase in the number of small-scale industries with a corresponding increase in welding activities. For example, in 2017, the *Mwanza Regional Investment Guide* estimated that approximately 13% of the total small-scale industries were involved in welding activities [19].

Although welding activities employ a significant number of people in Tanzania, there is limited information on the welders’ knowledge of, and adherence to, safety practices as outlined in Tanzanian OSH and how this affects workers’ exposure to occupational health and hazards. Evolving evidence from other African countries reveals limited knowledge about and use of PPEs among welders in the workplace. For example, a study conducted in Kaduna, Nigeria, found that only 34.2% of welders used one or more of PPE during welding activities [20]. Another study from Ethiopia found that only 32.3% of welders were knowledgeable of occupational safety practices [21], whereas 62.6% of welders in Delta State reported awareness of OHS guidelines [22]. In Sokoto in Nigeria, 9.6% to 83.2% of welders never used PPE at all [23].

In addition to the low level of knowledge and limited use of PPE, informal welders do not benefit from training programs or oversight through regular OSHA and/or health officers from various municipals inspections. Most informal sector workers do not have formal training and lack access to training opportunities at the welding site. There is simultaneously a lack of comprehensive surveillance of occupational hazards and/or injuries in the informal sector and so the degree of workers environmental and occupational health risk is not well documented.

To address this gap, our study of informal welders evaluated the relationship of OHS knowledge, training, and site inspection to health and safety guidelines adherence, including the use of PPE. This cross-sectional study of 219 adult informal welders at 70 informal welding sites in Mwanza City, Tanzania aimed to: 1) capture knowledge and awareness of occupational risks and safety precautions, training, and self-reported work-related injuries and illness and 2) observe worker use of personal protective equipment and site safety. We hypothesized that knowledge, awareness, and site inspections would improve use of PPE and that improved safety and site inspections would reduce self-reported injuries and illness.

## 2. Materials and methods

### 2.1. Study design and setting

The cross-sectional study surveyed 219 adult (older than 18 years of age) informal welders working at 70 informal electric welding sites across Mwanza City, in northwestern Tanzania on Lake Victoria from June 2021 –May 2022. Mwanza City is comprised of two main administrative districts: Nyamagana District (with 12 wards) and Ilemela District (with 9 wards). According to the 2012 national population census, Mwanza city had a population of 706,453 (342,530 male and 363,923 female) [18, 24].

### 2.2. Sampling and recruitment

The Mwanza City Council (MCC) of Social Affairs maintains an inventory of groups of welders who register for social affairs benefits, including getting loan support from the government. They do not necessarily register as a site (though it is possible) and welders do not register as individuals. The group is formal, but individuals who are informal workers cannot apply directly as an individual. This functions to access capital since informal workers cannot typically access formal bank loans due to extensive requirements for documentation, including financial stability. The organized group that registers determines what the loan request is, for example improving the welding site, and the group takes social accountability.

Using the inventory of registered welding groups, areas with the highest concentrations of welding activities were identified to target sampling. Then, within each of these areas, sites were identified by walking along the street and stopping at each welding site. All welders who were present at that time at a specific site were invited to participate. Participation was voluntary; a total of 219 adult welders signed the consent forms and participated in the study.

### 2.3. Data collection

A semi-structured questionnaire was adapted from a previous study that examined prevalence and determinants of occupational injuries among welders in small scale metal workshops in Wakiso District, Uganda (2021) and incorporating information from the *Tanzanian OSHA Fifth Schedule*, *sections 63*,*62*,*61*, *and 95 of OSH Act no 5 of 2003 and 2015 rules* [15, 16]) (*See S1 Table*). The survey also collected information on socio-demographic characteristics, including education level, history of working on welding sites, working hours, and age. Respondents answered a range of questions about, 1) knowledge of existing safety regulatory guidelines, 2) knowledge of occupational safety practices, 3) knowledge of occupational risks, and 4) perceived relevance of occupational safety practices (PRSP). Individual welders were asked about the area of the welding process and site where they worked for more than 90% of their time at the welding site. Composite scores were created for occupational risk, (three yes/no questions), knowledge of occupational safety practices (10 questions, very important/important/not important), PRSP (four questions, relevant/not relevant) (*See S2 Table*). A score for a given category was computed as follows:

$$Composite_{X}=\frac{\sum {\text{N}}_{X_{i}}}{\sum_{\text{i}=1}^{\text{K}}N_{X}}$$ (1)

where $\sum {\text{N}}_{X_{i}}$ is the sum of the number of questions with response given by participant *i*, whereas *$\sum_{\text{i}=1}^{\text{K}}N_{X}$* is the total number of questions for a given category (e.g. PRSP) considered in our checklists. No composite value was computed for awareness of occupational regulatory guidelines, as this category had one question with a binary response.

To enhance quality of the results, the survey was piloted to ten welders outside of the study area to inform the refinement of the survey and ensure questions were clear to the respondent. The questionnaire was administered orally in-person with responses recorded on paper during a face-to-face interview at the job site. In addition, a pre-designed checklist was used by the data collector to capture information on adherence to OSH guidelines. The inspection checklist, adapted from the *Tanzanian OSHA Fifth Schedule*, *sections 63*,*62*,*61*, *and 95 of OSH Act no 5 of 2003 and 2015 rules* [15, 16], captured workers’ use of PPE (safety boots, safety goggle, respiratory or dust mask, and gloves) during welding activities based on the type of activity and associated required PPE as specified by the American Safety and Health Fact Sheet for Welding and Cutting [14]. The checklist also captured site hazards, including fire risks, lack of grounding, water on-site, sanitary conveniences, washing facilities, lack of first aid facilities, protection against dust and fumes and protection of eyes in welding (*see S3 Table*). Binary response for each PPE use: yes = 1; no = 0. The composite score for the PPE using the same equation as for the survey questions. The use of equipment was recorded as yes/no, as was the number of times a safety violation was observed.

### 2.4. Ethical considerations

Ethical clearance was obtained from the joint Catholic University of Health and Allied Science (CUHAS) and Bugando Medical Center Ethics and Research Review Committee (Certificate no *CREC/476/2021*). Permission to conduct the research was obtained from the Mwanza Regional Administrative Secretary Office (*Ref*. *NO*. *FA*.*137/264/01J/23*), Mwanza City Council (*Ref*. *No*. *T40/7/122*) and Ilemela District Council (*Ref*. *IMC/T*.*40/7/VIL*.*IV/15*). Written informed consent was obtained from all informal welders who participated in this study. Participation in this study was voluntary. Participants signed an informed consent form (written in Kiswahili, the primary language of most of the population of Tanzania) prior to data collection (*See S1 Checklist*). In instances where individual welders had low literacy, a research assistant read and reviewed the consent form with the prospecting participants and a thumbprint was obtained indicating their consent. In all cases, an impartial witness from the local community was present during the informed consent process.

### 2.5. Statistical analysis and data management

Data cleaning and analysis were performed using SPSS Statistics 23 (SPSS Inc., Chicago IL) and RStudio version 4.2.1. A preliminary exploration of the data was done to check for missing values, duplicates, and unusual observations before analysis. In descriptive statistics, continuous variables were summarized using median and interquartile range while categorical variables were summarized using frequency and percentages.

A generalized linear model (GLM) was used for three separate analyses. The first assessed the association between knowledge of risks, knowledge of safety measures, site inspection, training in OHS, and educational level (predictors), and the use of PPE (response variable). Next, the impacts of knowledge of safety, knowledge of risk and PPE were used to predict the odds of occupational hazards. Since occupational hazards, including fire explosions, radiation exposure, electric shocks, and head/hand cuts were all binary outcomes, the binary logistic function in GLM was used to compute the odds. Lastly, the association between knowledge of risks and safety measures, site inspection, age, duration at the site, educational level (predictors), and welders’ PRSP (response variable) was examined. Association was considered statistically significant at *p*<0.05.

## 3. Results

### 3.1. Descriptive statistics

**Fig 1:**
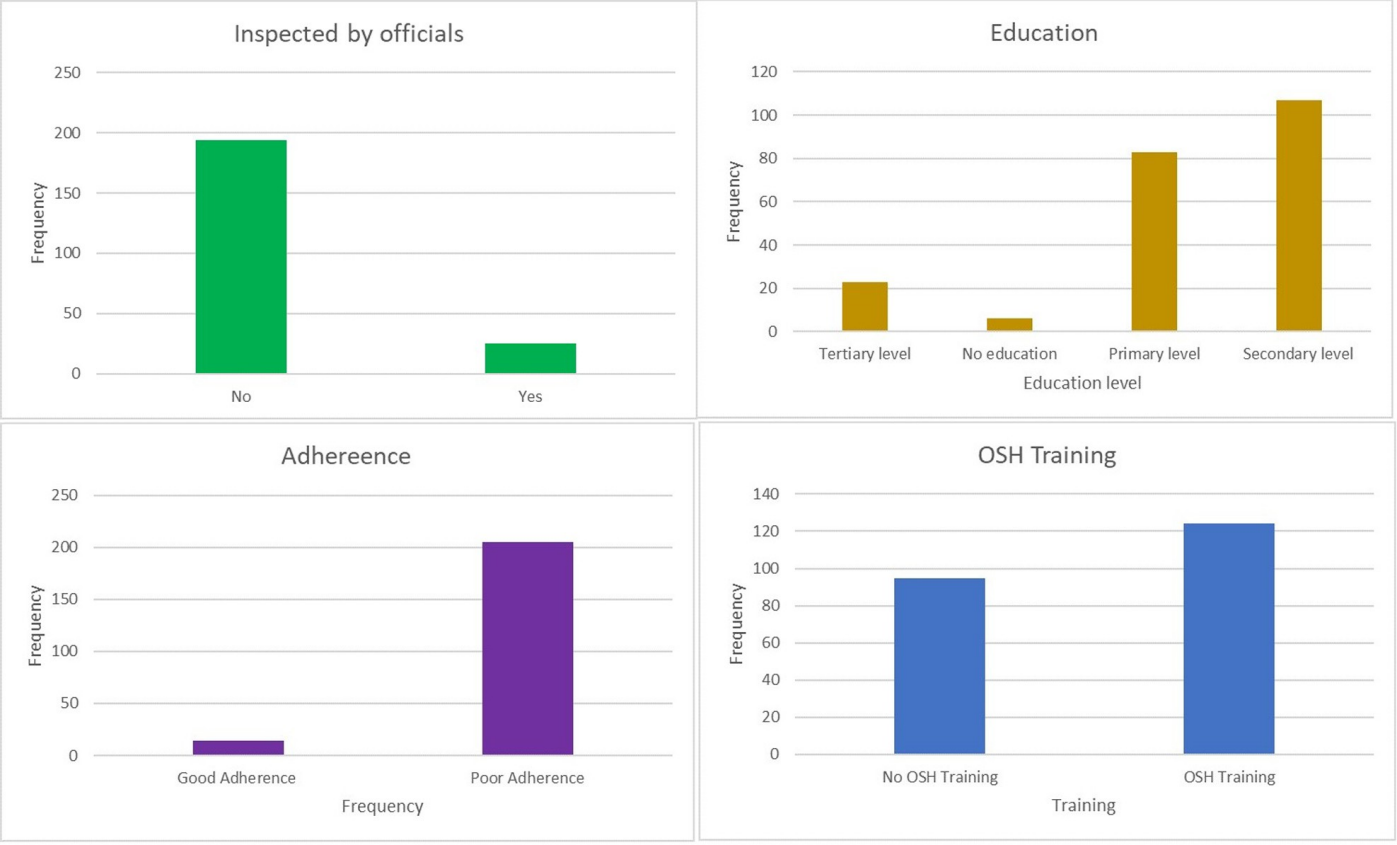
Characteristics of informal welders (*OSH training*, *adherence*, *inspection and education*).

  Table 1 and Fig 1 summarizes the background and characteristics of informal welders, along with the composite scores for knowledge, safety, and adherence (*See* S1 Data for extended data information). All of 219 informal welders were men and nearly half (49%) were aged between 25–34 years (median 29, IQR 25–34). The median duration of working at the site was 4 years (IQR: 2–6 years). Most (97.3%) of the informal welders had completed at least primary education and 10.9% knew of OSH laws and regulations. Although 57.1% of the informal welders reported having at least one OSH training, only 11.4% reported being inspected by government authorities on-site (Fig 1). Regarding training, 32% of welders had formal vocational and technical training, whereas 68% received their training onsite. Only 37% of the informal welders had knowledge of occupational safety practices and 93.6% had poor adherence to OSH guidelines. A majority (69%) had a high perception of PRSP. The median knowledge of occupational risk score was 3 (IQR 2–3), knowledge of safety measures 17 (IQR 16–18), PPE use score 5 (IQR 4–5) and PSPP 4 (IQR 3–4).

**Table 1:** Background and characteristics of the informal welders (N = 219).

**Variables**	**n**	**%**
**Age**		
19–24	72	32.88
25–34	107	48.86
35–69	40	18.26
Median age (IQR)	28 (24–32)	
**Education level**		
No formal education	6	2.74
At least primary education	213	97.26
**District**		
Nyamagana	38	17.35
Ilemela	181	82.65
Jobs Involved**		
Welding only	73	33.33
Cutting and hammering only	34	15.53
Painting only	12	5.48
Involved in all activities	100	45.66
**Working experience**		
Less 1 year	47	21.46
1–5 years	100	45.66
Above 5 years	72	32.88
Median duration at the site (IQR)	4(2–6)	
**Received any training**		
The Vocational Training Center (VTC)	71	32.42
Site Training	148	67.58
**Safety training gained**		
At least one safety training	125	57.08
None	94	42.92
**Inspection from gov’t authorities**		
Yes	25	11.42
No	194	88.58
**Knowledge of OHS laws**		
Yes	24	10.96
No	195	89.04
**Safety practices knowledge rating**		
Has knowledge (50% and above)	81	36.99
Do not have knowledge (<50%)	138	63.01
**Perceived safety practice status**		
Good	150	68.49
Poor	69	31.51
**Adherence to OHS guidelines**		
Good (50% and above)	14	6.39
Poor (<50%)	205	93.61

Note: **The most common activities or job reported to be carried out by respondents by 90% of their time at the welding workshop.

Reported injury among participants reveals a significant occupational health burden. A large percentage of the participants reported experiencing electric shock (61.64%; n = 35), fire outbreaks (57.9%; n = 49), respiratory complications (64.8%; n = 142), and nasal problems (73.5% n = 161).

### 3.2. Factors associated with PPE use

In the unadjusted model, knowledge of occupational risks (cβ = 1.335; 95% CI: 1.184, 1.506, *p*<0.001), site inspection (cβ = 1.103; 95% CI: 1.003, 1.212, *p* = 0.043) and having training in OSH (cβ = 1.089; 95% CI: 1.025, 1.157, *p* = 0.006) were significantly positively associated with the use of PPE, while knowledge of safety measures (cβ = 1.149; 95% CI: 0.896, 1.473, *p* = 0.273) was not statistically significant, even though it increased PPE use by 14%. Having primary (cβ = 0.871; 95% CI: 0.762-.995, *p* = 0.042) and secondary-level education (cβ = 0.945, 95% CI: 0.830–1.075, *p* = 0.758) were associated with less use of PPE, even though the relationship was not statistically significant for secondary school. Furthermore, duration working at the site (cβ = 1.003; 95% CI: 0.996, 1.011, *p* = 0.428) and age (cβ = 1.000; 95% CI: 0.996, 1.005, *p* = 0.809) were not significantly associated with PPE use (Table 2).

**Table 2:** Crude and adjusted model for (1) factors associated with PPE use, (2) factors associated with PSPP and (3) associated factors for occupational hazards.

	Crude Model			Adjusted Model		
Variable	Coefficient	95% CI (LL-UL)	*p*-value	Coefficient	95% CI (LL-UL)	*p*-value
***Factors associated with PPE use***.						
Knowledge of occupational risks	1.335	1.184–1.506	<0.001***	1.305	1.143–1.491	<0.001***
Site inspection	1.103	1.003–1.212	0.043*	0.928	0.872–0.988	0.019*
Having training in OSH	1.089	1.025–1.157	0.006**	1.0753	1.147–1.016	0.029*
Knowledge of safety measures	1.149	0.896–1.473	0.273	1.112	0.881–1.404	0.372
**Education**						
Post-secondary education	0.962	0.825–1.120	0.616	0.942	0.788–1.127	0.514
Secondary-level education	0.945	0.830–1.075	0.387	0.925	0.792–1.082	0.331
Having primary	0.871	0.762-.995	.042*	0.887	0.758–1.040	0.139
Having no education	Ref	‐‐‐‐	‐‐‐‐	ref	‐‐‐‐‐‐	‐‐‐‐‐‐
Duration working at the site	1.003	0.996–1.011	0.428	1.008	0.999–1.017	0.066
Age of Welders	1.000	0.996–1.005	0.809	0.997	0.993–1.000	0.042*
***Factors associated with perceived relevance of safety practices (PRSP*** *)*						
Knowledge of occupational risks	1.865	1.416, 2.472	0.001**	1.574	1.404, 1.756	<0.001**
Knowledge of occupational safety	1.657	1.475, 1.861	0.001**	1.628	1.34, 1.978	<0.001**
Site inspection (Yes is ref)	0.953	0.87, 1.037	0.245	0.994	0.923, 1.070	0.873
Age of welders	1.006	1.003, 1.009	<0.001**	1.001	0.998, 1.004	0.527
Duration at site	1.01	1.004, 1.015	<0.001**	1.003	0.998, 1.008	0.226
**Education**						
Post-secondary education	1.199	0.991, 1.449	0.065*	1.195	1.023, 1.397	0.025*
Secondary-level education	1.202	1.010, 1.430	0.038*	1.187	1.031, 1.368	0.017*
Having primary	1.147	0.963, 1.367	0.124	1.179	1.021, 1.361	0.025*
Having no education	Ref	‐‐‐‐	‐‐‐‐‐	‐‐‐	‐‐‐‐	‐‐‐‐
*Risk factors of occupational hazards*						
head cuts						
	Coefficient (OR)					
PPE use	0.038	0.008, 0.179	0.001**	0.052	0.010, 0.279	<0.001**
PRSP	0.067	0.01, 0.299	<0.001**	0.761	0.565, 1.026	0.073
Knowledge of occupational risks	0.157	0.044, 0.566	0.005**	0.577	0.120, 2.771	0.492
Knowledge of safety measures	0.002	0.000, 0.068	<0.001**	0.009	0.000, 0.267	0.006**
Electric shocks						
PPE use	0.042	0.007, 0.275	0.001**	0.019	0.002, 0.164	<0.001**
PRSP	0.756	0.056, 10.273	0.833	0.362	0.078, 1.673	0.193
Knowledge of occupational risks	0.328	0.024, 4.570	0.407	0.076	0.002, 2.583	0.152
Knowledge of safety measures	0.065	0.006, 0.770	0.03*	0.548	0.002, 197.126	0.841
fire explosions						
PPE use	0.060	0.015, 0.246	<0.001**	0.105	0.023, 0.475	0.003**
PRSP	0.043	0.009, 0.199	<0.001**	0.075	0.018, 0.314	<0.001**
Knowledge of occupational risks	0.107	0.029, 0.395	<0.001**	0.548	0.102, 2.936	0.483
Knowledge of safety measures	0.065	0.006, 0.770	0.03*	0.215	0.012, 3.748	0.292
Radiation exposure						
PPE use	0.089	0.019, 0.426	0.002**	0.127	0.023, 0.708	0.019*
PRSP	0.216	0.022, 2.078	0.185	0.738	0.136,0.934	0.040*
Knowledge of occupational risks	0.107	0.029, 0.395	<0.001**	0.263	0.037, 1.860	0.181
Knowledge of safety measures	0.065	0.006, 0.770	0.03*	1.260	0.031, 50.931	0.902
Hand cuts						
PPE use	0.601	0.121, 3.034	0.543	0.747	0.138, 4.052	0.735
PRSP	0.257	0.049, 1.346	0.108	0.783	0.625, 0.98	0.034*
Knowledge of occupational risks	0.673	0.144, 3.146	0.615	0.608	0.08, 4.440	0.624
Knowledge of safety measures	0.223	0.014, 3.525	0.287	0.407	0.018, 9.166	0.572

In our adjusted model, an increased knowledge of occupational risks was associated with a 31% increased use of PPE (aβ = 1.305, 95% CI: 1.143, 1.491; *p*<0.001). Education had no association with PPE use. Increased knowledge of occupational safety measures increased the use of PPE by 11% (aβ = 1.112, 95% CI: 0.881, 1.404; *p* = 0.372) even though the association was also not statistically significant. Additionally, we found that = those with training had a 7.5% increase in PPE use (aβ = 1.0753, 95% CI: 1.147–1.016, *p* = 0.029) over those with no training. Further, sites with no government safety inspection had a 7.2% decline in use of PPE (aβ = 0.928; 95% CI: 0.872, 0.988, *p* = 0.019).

### 3.3. Factors associated with perceived relevance of safety practices (PRSP)

In the unadjusted model, we found that increased knowledge of occupational risks was associated with an 87% (cβ = 1.865, 95% CI: 1.416, 2.472, *p* = 0.001) increase in PRSP and an increased knowledge of occupational safety measures was associated with a 66% (cβ = 1.657, 95% CI: 1.475, 1.861; *p*<0.001) increase in PRSP. Those sites with no government safety inspection had a 5% reduction in PRSP (cβ = 0.953, 95% CI: 0.87, 1.037, *p* = 0.245) even though the association was not statistically significant. Furthermore, age (cβ = 1.006, 95 CI: 1.003, 1.009; *p*<0.001) and longer working duration at the welding sites (cβ = 1.01, 95% CI: 1.004, 1.015; *p*<0.001) both increased PRSP by 0.6% and 10% respectively. Additionally, as compared to having no education, having primary (cβ = 1.147; 95% CI: 0.963, 1.367, *p* = 0.124), secondary (cβ = 1.202; 95% CI: 1.010, 1.430, *p* = 0.038) and post-secondary education (cβ = 1.199; 95% CI: 0.991, 1.449, *p* = 0.062) was positively associated with PRSP.

In the adjusted model, increased knowledge of occupational risks was linked with a 57% (aβ = 1.57, 95% CI: 1.404, 1.756; *p*<0.001) increase in PRSP. A higher knowledge of occupational safety measures was associated with a 63% (aβ = 1.628, 95% CI: 1.34, 1.978; *p*<0.001) increase in PRSP. However, age 0.1% (aβ = 1.001, 95% CI: 0.998, 1.004; *p* = 0.527) and duration at site 0.3% (aβ = 1.003, 95% CI: 0.998, 1.008; *p* = 0.226) were not statistically significantly associated with PRSP. Lastly, as compared with having no education, having at least primary (aβ = 1.142; 95% CI: 0.99, 1.310, *p* = 0.059), secondary (aβ = 1.159; 95% CI: 1.012, 1.327, *p* = 0.033) and college level education (aβ = 1.172; 95% CI: 1.010, 1.359, *p* = 0.036) was associated with higher PRSP.

### 3.4. Risk factors of occupational hazards

In the crude model, PPE use was statistically significantly associated with reduced odds of head cuts (COR = 0.038; 95% CI: 0.008, 0.179, *p*<0.001), electric shocks (COR = 0.042; 95% CI: 0.007, 0.275, *p*<0.001), fire explosions (COR = 0.060; 95% CI: 0.015, 0.246, *p*<0.001), and radiation exposure (COR = 0.089; 95% CI: 0.019, 0.426, *p* = 0.002), but not significantly associated with hand cuts (COR = 0.60; 95% CI: 0.121, 3.034, *p* = 0.543). Furthermore, higher PRSP was associated with decreased odds of electric shocks (COR = 0.756; 95% CI: 0.056, 10.273, *p* = 0.833), radiation exposure (COR = 0.216; 95% CI: 0.022, 2.078, *p* = 0.185), fire explosions (COR = 0.043; 95% CI: 0.009, 0.199, *p*<0.001), hand cuts (COR = 0.257; 95% CI: 0.049, 1.346, *p* = 0.108) and head cuts (COR = 0.067; 95% CI: 0.01, 0.299, *p*<0.001), even though none was statistically significant. Additionally, increase knowledge of occupational risks was associated with reduced odds of hand cuts (COR = 0.673, 95% CI: 0.144, 3.146, *p* = 0.615), head cuts (COR = 0.157; 95% CI: 0.044, 0.566, *p* = 0.005), electric shocks (COR = 0.328; 95% CI: 0.024, 4.570, *p* = 0.407), fire explosions (COR = 0.107; 95% CI: 0.029, 0.395, *P*<0.001), and radiation exposure (COR = 0.29, 95% CI: 0.064, 1.377, *p* = 0.121), even though only fire explosions was statistically significant. Increased knowledge of safety measures was significantly associated with lesser odds of fire explosions (COR = 0.065; 95% CI: 0.006, 0.770, *p* = 0.03) and head cuts (COR = 0.002; 95% CI: 0.000, 0.068, *p*<0.001), but was not significant for electric shocks (COR = 1.086; 95% CI: 0.012, 96.096, *p* = 0.97), radiation exposure (COR = 0.32; 95% CI: 0.015, 6.732, *p* = 0.463) and hand cuts (COR = 0.223; 95% C: 0.014, 3.525, *p* = 0.287).

In the adjusted model, the use of PPE was found to have a significant impact on the odds of occupational hazards. For example, PPE use was associated with a significant reduction in head cuts (AOR = 0.052; 95% CI: 0.010, 0.279, *p*<0.001), electric shocks (AOR = 0.019; 95% CI: 0.002, 0.164, *p*<0.001), fire explosions (AOR = 0.105; 95% CI: 0.023, 0.475, *p* = 0.003), and radiation exposure (AOR = 0.127; 95% CI: 0.023, 0.708, *p* = 0.019), but not significant with hand cuts (AOR = 0.747; 95% CI: 0.138, 4.052, *p* = 0.735). Increased PRSP was associated with a significantly lesser likelihood of experiencing electric shocks (AOR = 0.362, 95% CI: 0.078, 1.673; *p* = 0.193), radiation exposure (AOR = 0.738, 95% CI: 0.136,0.934, *p* = 0.04), fire explosion (AOR = 0.075, 95% CI: 0.018, 0.314; *p*<0.001), hand cuts (AOR = 0.783, 95% CI: 0.625, 0.98; *p* = 0.034) and marginally significant with head cuts (AOR = 0.761, 95% CI: 0.565, 1.026; *p* = 0.073). There was no association between knowledge of occupational risks and radiation exposure (AOR = 0.263, 95% CI: 0.037, 1.86, *p* = 0.181), electric shocks (AOR = 0.076; 95% CI: 0.002, 2.583, *p* = 0.152), fire explosions (AOR = 0.548; 95% CI: 0.102, 2.936, *p* = 0.483), hand cuts (AOR = 0.608; 95% CI: 0.08, 4.440, *p* = 0.624) and head cuts (AOR = 0.577; 95% CI: 0.120, 2.771, *p* = 0.492). Similarly, there was no association between knowledge of safety measures and hand cuts (AOR = 0.407; 95% CI: 0.018, 9.166, *p* = 0.572), head cuts (AOR = 0.009; 95% CI: 0.000, 0.267, *p* = 0.006), electric shocks (AOR = 0.548, 95% CI: 0.002, 197.126, *p* = 0.841), fire explosion (AOR = 0.215; 95% CI: 0.012, 3.748, *p* = 0.292), and radiation exposure (AOR = 1.260; 95% CI: 0.031, 50.931, *p* = 0.902).

## 4. Discussion

Workplace safety is integral to industrial prosperity, but unfortunately most informal welders are subjected to substantial occupational health risks. Especially for informal welders who know little about risks and safety practices or the benefits of the use of PPE, poor health outcomes and injury increase substantially [17]. Our current study revealed that many of the informal welders had poor knowledge of workplace safety practices and limited knowledge of OHS guidelines set by the Tanzanian OSHA. This is consistent with previous studies from Nigeria and Tanzania where 64.9% of self-employed electric arc welders had poor knowledge of OSH and 55.7% had limited knowledge of OSH rules and regulations respectively [25, 26]. Most (87.1%) of the welders in this study had no formal training on safety measures and 37% rarely used PPE when performing their duties, which is consistent with previous studies from Tanzania and Nigeria [25, 26].

Poor knowledge of safety practices can be attributed to the modality of training. For example, most of the respondents in our study received their training informally through onsite training (e.g., via apprenticeship) and so rely extensively on their co-workers, who are likely untrained as well, for information regarding safety practices and risks associated with welding activities. However, contrary to our findings, studies from Jinja-Uganda and Ethiopia noted that 50% and 56% of informal welders were knowledgeable about safety practices respectively [21, 27]. The differences in the level of knowledge regarding safety practices between our findings and those from Kenya and Uganda may be attributed to variations underlying the sociodemographic backgrounds of the study subjects (e.g., education level) or possibly the (in)effectiveness of OSH guidelines regulation.

Research from Kenya and India reported a significant association between increased knowledge of occupational risks, knowledge of safety practices, degree of OSH law enforcement, and level of education with the adoption of safety practices including the use of PPE [28, 29]. Our study is in alignment with these studies and further substantiates the need for formal training programs. Further, the importance of inspections from government oversight to enforce OSH, using this as an opportunity to increase awareness, substantially improves PPE use and decreases injuries.

The informal nature of training and almost no inspection from enforcement officials contributes to the majority (94%) of welders not adhering to the established OSH guidelines and by extension less use of PPE. This is also supported by a study from Delta State in Nigeria where increased use of PPE (62.6%) was associated with adherence to the OSH guidelines due to constant inspection at welding sites [22]. Crucially, although the majority (69%) of the informal welders had a positive perception regarding the relevance of safety practices, observing these practices, including the use of PPE, was more challenging for them. As another key finding in our study, higher levels of occupational risks/safety knowledge and heightened perception of importance of safety practices led to higher use of PPE and resulted in reduced injury. Welders who use PPE were less likely to experience fire explosions, radiation exposures, hand cuts, head cuts, and electric shocks. Providing the needed incentive, creating more awareness about occupational health, along with enforcement/inspection, would go a long way to make significant improvements in adopting safety practices.

Without the proper use of PPE, welders may be exposed to respirable dust which has the potential to penetrate the alveoli and result in serious respiratory health problems [30]. PM_2.5_, or respirable dust, can pass through the nasal passage and penetrate the alveoli and may result in serious respiratory health effects depending on the content of the welding fumes [30, 31]. Further, welding fumes contain toxic chemicals, such as cadmium, chromium, manganese, and nickel from the base metals used in welding. These have been associated with lung cancer and neurological effects, especially upon prolonged exposure [32, 33]. A previous study in Dar es Salaam, Tanzania reported an association between metal fume (PM_2.5_ 6.57±2.50mg/m^3^) and respiratory symptoms among small-scale welders [9]. Prolonged exposure can result in serious chronic and debilitating respiratory health conditions. Taken together, the health and safety of informal welders can be improved immensely by proper use of PPE; training, education, policies, and site inspections together increase adherence.

### Study limitations

Due to COVID-19 restrictions and the need for adequate social distancing, we could not conduct a lung functioning test to understand the effects of air pollution, nor did we assess noise-induced hearing loss. Further, health effects and symptoms were self-reported and did not have a confirmed diagnosis. We did not test for the chemical composition of welding fumes, like cadmium, chromium, manganese, and nickel, or consider potential health outcomes from these exposures.

## 5. Conclusion

Evidence shows that increasing OSH education campaigns, enforcing (encouraging) welders to use PPE and safety practices, inspections from government officials, and providing the right incentive can improve the working conditions for informal welders. Less knowledge about safety practices contributes to reduced usage of PPE, which in turn results in more workplace injury. Continued worker health training programs that aim to increase knowledge of welding occupational risk and safety practices, refinement, and enforcement of OSH to directly address welding, and inspection by government officials will improve the health and safety for informal welders. Coordinated intervention efforts between the Tanzanian government, academia, NGOs, and the welding site managers can have extensive worker health benefits.

## Supporting information

S1 Checklist Inclusivity in global research. (DOCX)

S1 Data De-identified data and detailed information regarding study participants. (CSV)

S1 Table Questionnaires on Assessment of informal welder’s knowledge on safety practices and adherence to occupational health and safety guidelines. (DOCX)

S2 Table Composite variables used for constructing perceived safety practices (response variables), knowledge of safety practices, and risk scores (predictors). (DOCX)

S3 Table Checklist on adherence to OHS Guidelines requirements as per OHS ACT 5 OF 2003; and its regulations. (DOCX)

## References

[1] WHO/ILO: Almost 2 million people die from work-related causes each year. Occupational safety and health, 2021. Available at, https://www.ilo.org/global/about-the-ilo/newsroom/news/WCMS_819705/lang—en/index.htm

[2] Takala J, Hämäläinen P, Saarela KL, Yun LY, Manickam K, Jin TW, et al. Global estimates of the burden of injury and illness at work in 2012. J Occup Environ Hyg. 2014;11(5):326–37. doi: 10.1080/15459624.2013.863131 24219404 PMC4003859

[3] Mrema EJ, Ngowi AV, Mamuya SH. Status of Occupational Health and Safety and Related Challenges in Expanding Economy of Tanzania. Ann Glob Health. 2015 Jul-Aug;81(4):538–47. doi: 10.1016/j.aogh.2015.08.021 26709286

[4] Rongo LM, Barten F, Msamanga GI, Heederik D, Dolmans WM. Occupational exposure, and health problems in small-scale industry workers in Dar es Salaam, Tanzania: a situation analysis. Occup Med (Lond). 2004 Jan;54(1):42–6. doi: 10.1093/occmed/kqh001 . 14963253

[5] Loewenson R. Globalization and occupational health: a perspective from southern Africa. Bull World Health Organ. 2001;79(9):863–8. Epub 2001 Oct 23. ; PMCID: PMC2566652. 11584735 PMC2566652

[6] Vendan SA, Gao L, Garg A, Kavitha P, Dhivyasri G, Rahul SG. Codes and Safety Standards During Welding. In: Interdisciplinary Treatment to Arc Welding Power Sources. Springer; 2019. p. 209–29. 10.1007/978-981-13-0806-2_7

[7] Parmar RS. Welding processes and technology. Khanna Publishers; 2001.

[8] Gonser M, Hogan T. Arc welding health effects, fume formation mechanisms, and characterization methods. Arc welding. 2011;299–320. Available at, https://cdn.intechopen.com/pdfs/25071/InTech-Arc_welding_health_effects_fume_formation_mechanisms_and_characterization_methods.pdf

[9] Meena GG. Assessment of occupational exposure to metal fumes and associated respiratory health symptoms among small scale welders in Dar es salaam. Muhimbili University of Health and Allied Sciences, Master Thesis. 2017. Available at, http://dspace.muhas.ac.tz:8080/xmlui/bitstream/handle/123456789/2702/MEENA%20DISSERTATION.pdf

[10] Zgambo J. Occupational hazards and use of personal protective equipment among small scale welders in Lusaka, Zambia. Occupational health. The University of Bergen. Norway. Master Thesis. 2015. Available at, https://core.ac.uk/download/pdf/30899528.pdf

[11] Alli BO, Benjamin O. Fundamental principles of occupational health and safety. 2nd Edition Vol. 39, Choice Reviews Online. ILO, Geneva. 2002. 39-3997-39–3997 p. Available at, https://www.ilo.org/global/publications/ilo-bookstore/order-online/books/WCMS_093550/lang—en/index.htm

[12] Sabitu K, Iliyasu Z, Dauda MM. Awareness of occupational hazards and utilization of safety measures among welders in Kaduna metropolis, northern Nigeria. Ann Afr Med. 2009 Jan-Mar;8(1):46–51. doi: 10.4103/1596-3519.55764 19763007

[13] Tadesse S, Bezabih K, Destaw B, Assefa Y. Awareness of occupational hazards and associated factors among welders in Lideta Sub-City, Addis Ababa, Ethiopia. J Occup Med Toxicol. 2016 Apr 5;11:15. doi: 10.1186/s12995-016-0105-x 27051458 PMC4820982

[14] American Welding Society. Safety and Health Fact Sheet No. 33: Personal Protective Equipment (PPE) for Welding and Cutting, Doral, Florida 33166. 2014. Available from, https://app.aws.org/technical/facts/FACT-33_2014.pdf

[15] United Republic of Tanzania (URT). Occupational Health and Safety Act, 2003 (No. 5 of 2003). United Republic of Tanzania: Government Publisher; Dar Es Salaam. 2003. p. 50,66–69,82–83. Available at, https://www.ilo.org/dyn/natlex/docs/ELECTRONIC/64739/61555/F481973607/TZA64739.pdf

[16] United Republic of Tanzania (URT). National Occupational Health and Safety policy. Ministry of Labour and Employment. United Republic of Tanzania: Government Publisher; Dar Es Salaam. 2009;1–30. Available at, https://www.ilo.org/wcmsp5/groups/public/—ed_protect/—protrav/—safework/documents/policy/wcms_326526.pdf

[17] Asgedom AA, Bråtveit M, Moen BE. Knowledge, attitude, and practice related to chemical hazards and personal protective equipment among particleboard workers in Ethiopia: a cross-sectional study. BMC Public Health. 2019 Apr 27;19(1):440. doi: 10.1186/s12889-019-6807-0 31029126 PMC6487067

[18] Tanzania National Bureau of Statistics (TNBS), 2013. The 2012 Population and Housing Census: Population Distribution by Administrative Areas. United Republic of Tanzania. Dar es salaam. March 2013. Accessed on January 2018, Available at, http://www.tzdpg.or.tz/fileadmin/documents/dpg_internal/dpg_working_groups_clusters/cluster_2/water/WSDP/Background_information/2012_Census_General_Report.pdf

[19] United Republic of Tanzania 2017. Mwanza Investment Guide in the Support of United Nations Development Programme (UNDP) and Economic Social Research (ESRF). 2017;(SBN: 978-9976-5231-0-2). Available at, https://mwanza.go.tz/storage/app/uploads/public/5a1/c20/c64/5a1c20c641dbf041333499.pdf

[20] Sabitu K, Iliyasu Z, Dauda MM. Awareness of occupational hazards and utilization of safety measures among welders in Kaduna metropolis, northern Nigeria. Ann Afr Med. 2009 Jan-Mar;8(1):46–51. doi: 10.4103/1596-3519.55764 19763007

[21] Beyene Gebrezgiabher B, Tetemke D, Yetum T. Awareness of Occupational Hazards and Utilization of Safety Measures among Welders in Aksum and Adwa Towns, Tigray Region, Ethiopia, 2013. J Environ Public Health. 2019 Jan 21;2019:4174085. doi: 10.1155/2019/4174085 30805014 PMC6360553

[22] Obarhoro OI, Nwufo CR, Nworu B, Ibe SNI, Iwuala CC, Ede A, et al. Compliance in the Use of Personal Protective Equipment by Welders in Delta State, Nigeria. International Journal of Research and Review. 2020;7(1):21–6.

[23] Awosan K, Makusidi M, Ibrahim M, Suleiman A, Magaji T, Mbatifuh F. Knowledge and Safety Practices Related to Exposure to Physical and Chemical Hazards among Welders in Sokoto, Nigeria. Asian Journal of Medicine and Health. 2017;9(1):1–11. doi: 10.9734/AJMAH/2017/3

[24] Tanzania National Bureau of Statistics. Tanzania Demographic and Health Survey and Malaria Indicator Survey (TDHS-MIS) ‐ Final Report 2015–16. Dar es Salaam, Tanzania. 2017. Available at https://dhsprogram.com/pubs/pdf/fr321/fr321.pdf

[25] Adewoye KR, Awoyemi AO, Ibirongbe DO, Babatunde OA, Ibrahim T. Knowledge on the health effects of welding smoke, use of ppe among electric-arc welders in ilorin south, north central Nigeria. Journal of Asian Scientific Research. 2013;3(9):924–32.

[26] Mushi L. Factors Affecting Adherence to Occupational Health and Safety Rules and Regulations in Improving Public Health in the Informal Sector Workers in Mabibo-Dar es Salaam. 2019;7(1). doi: 10.25215/0701.079

[27] Oluwole I, Nwanna K, Afolabi KK, Ademola SA, Aremu AB, Mujeeb S, et al. Determinants of compliance with the occupational health and safety practices among metal welders in Jinja municipality-Jinja district Uganda. Vol. 07, Medical Safety & Global Health. OIMICS International; 2018. doi: 10.4172/2574-0407/1000140

[28] Odhiambo ON, Peterson W, Isaac M. Knowledge about Physical Hazards and Use of Personal Protective Equipment Based on Demographic Characteristics and among Small Scale Welders in Nairobi City, Kenya. J Sci Res Rep. 2020;120–30. 10.9734/jsrr/2020/v26i630277

[29] Kumar SG, Dharanipriya A, Kar SS. Awareness of occupational injuries and utilization of safety measures among welders in coastal South India. Int J Occup Environ Med. 2013 Oct;4(4):172–7. 24141865

[30] Hygienists AC of GI. 2005 TLVs and BEIs: Based on the Documentation of the Threshold Limit Values for Chemical Substances and Physical Agents & Biological Exposure Indices. In American Conference of Governmental Industrial Hygienists; 2005.

[31] Hariri A, Md Yusof MZ, Leman AM. Sampling method for welding fumes and toxic gases in malaysian small and medium enterprises (SMEs). Energy and Environment Research. 2012;2(2):1–8. doi: 10.5539/EER.V2N2P13

[32] Wang XL, Yang YJ, Ding MY, Quan ZJ, Zhao XG, Nie J, et al. [Effect of heavy metals exposure on neurobehavioral function in welders]. Zhonghua Lao Dong Wei Sheng Zhi Ye Bing Za Zhi. 2008 Nov;26(11):641–4. Chinese. 19467179

[33] Rana HK, Akhtar MR, Ahmed MB, Lio P, Quinn JMW, Huq F, et al. Genetic effects of welding fumes on the progression of neurodegenerative diseases. Neurotoxicology. 2019 Mar;71:93–101. doi: 10.1016/j.neuro.2018.12.002 30571986

