# Supplementary material for: Informal welders’ occupational safety and environmental health risks in northwestern Tanzania
Source: PLOS Glob Public Health. 2024 Feb 28;4(2):e0002923. doi: 10.1371/journal.pgph.0002923 (PMC10901300; doi:10.1371/journal.pgph.0002923)
Supplement: S1 Table — (DOCX) [file pgph.0002923.s003.docx]

**S1 Table:** Questionnaires on Assessment of informal welder’s knowledge on safety practices and adherence to occupational health and safety guidelines

**SECTION A: Socio-Demographic and General Information (***Please tick (√) where appropriate)*

| A1 | Sex of the welder? | [ ] Male  [ ] Female |
| --- | --- | --- |
| A2 | How old are you? | Mention your age………… |
| A3 | What is your duty on this site? | [ ] Welder  [ ] cutting  [ ] Painter  [ ] Owner of site  [ ] Other………………… |
| A4 | What is the highest level of education attained? | [ ] Primary  [ ] Secondary  [ ] Certificates  [ ] Degree |
| A5 | How were you trained to become a welder? | [ ] VTC  [ ] Apprenticeship  [ ] other……….............. |
| A6 | Where are any of the following covered in your training to become a welder? | [ ] Occupational Health and Safety issues  [ ] hazard identification  [ ] Health affects  [ ] Use of Personal Protective Equipment |
| A7 | How long have you been working in the welding industry? | Mention (Duration in months or Years) |
| A8 | Have you ever inspected/visited by Government Agency/Authority? | [ ]Yes  [ ] No  If yes answer the next question |
| A9 | What did the Governmental Agency/Authority advise you concerning safety practices in welding? | (Mention what advice they provided)  …………………………………………………  …………………………………………………. |

**Social-economic factors**

| A2-1 | This welding site is owned by whom? | [ ] Welder  [ ] Rented  Other mention………………… |
| --- | --- | --- |
| A2-2 | Who owns the welding machine you’re using? | [ ] Welder  [ ] Rented  Other explain…………………… |
| A2-3 | What are property do you own? | [ ] house  [ ] farm  [ ] land plots  [ ] Any means of transport  [ ] Other………………… |
| A2-4 | What means of transport do you always use to reach your office | [ ] private car  [ ] public transport  [ ] Bicycle  [ ] By foot  [ ] Other mention……….. |

**SECTION B-1: Knowledge on Occupational Health and Safety**

*(Please tick or fill the appropriate)*

| B-1(1) | Do you know any occupational health and safety laws and regulations in the country? | [ ] Yes  [ ] No  Mention the Act or regulation which you know |
| --- | --- | --- |
| B-1(2) | Why are you using electric welding other than other methods? | Explain……………………….……………………… |
| B-1(3)  a | Do you know the kind of metals that are involved in welding? | Mention: ………………………………. |
| B-1(3)  b | What consumables materials are used in welding? | Mention……………………………………………………………………… |
| B-1(4) | Do you know of any hazards due to welding cables and consumables kept in passageways? | [ ] Yes  [ ] No  If yes mention them………………………………………. |
| B-1(5) a | Do you know the importance of proper earthing clamps and cable connectors? | [ ] Yes  [ ] No |
| B-1(5) b | Do you check, inspect connections? | [ ] Yes  [ ] No  How often do you inspect in a month…………….  When was the last inspection……………………. |
| B-1(6) a | Does welding fumes, during welding have any health effect on your health? | [ ] Yes  [ ] No  If yes mention the health affects you encountered during welding? |
| B-1(6) b | How do you often protect yourself from welding fumes? | Explain………………………………………………………………………… |

**SECTION B-2: Occupational Safety Practices** ***(Please tick or fill the appropriate)***

| B-2(1) | How do you protect yourself from arc flash? | Explain………………………………… |
| --- | --- | --- |
| B-2(1) | How do you protect employees working nearby/passers from arc flash? | Explain………………………………… |
| B-2(2) | How can you avoid causes of fire risk at welding sites? | Mention………………………………………………………………………………………………………………………………………………………………… |
| B-2(3) | Where is the safer area to carry out the welding activities? | Explain………………………………………………………………………………………………………………………………………………………………… |
| B-2(4) | How important are the following measures for electric arc welding safety? | 1. To not coil or loop welding electrode cable around his or her body.   [ ]Very important [ ]somewhat important [ ]not important   1. To remove electrodes from the holders when not in use.   [ ]Very important [ ]somewhat important [ ]not important   1. To shut off power to the welder when no one is in attendance.   [ ]Very important [ ] somewhat important [ ]not important   1. To avoid welding where water is present.   [ ]Very important [ ]somewhat important [ ]not important   1. To dry hands before doing any welding.   [ ]Very important[ ] somewhat important [ ]not important   1. To disconnect electrical equipment immediately after use.   [ ]Very important [ ]somewhat important [ ]not important   1. To inspect and repair equipment. Work and electrode lead cables are frequently and inspected for wear and damage and replaced when necessary.   [ ]Very important [ ]somewhat important [ ] not important   1. To check the electrical safety of the rod holder or welding handpiece regularly and maintained or replaced as required.   [ ]Very important [ ]somewhat important [ ]not important |
| B-2(5) | How do you maintain general housekeeping at the site? | Explain………………………………………………………………………… |
| B-2(6) | What protective gears are you using to protect you from risks? | Mention: ……………………………………………………………………… |
| B-2(7) | Which of the following manual handling behaviors are involved in your daily works? | [ ] repetitive work  [ ] Fixed, sustained, rigid or awkward postures  [ ] Prolonged work, movement  [ ] Working with no rest/ break  [ ] Handling or reaching away from the body  [ ] Using high or sustained force  [ ] Repetitive works  [ ] Whole-body vibration or  [ ] Hand-arm vibration  [ ] Handling that goes on for too long without a break |
