# Supplementary material for: Informal welders’ occupational safety and environmental health risks in northwestern Tanzania
Source: PLOS Glob Public Health. 2024 Feb 28;4(2):e0002923. doi: 10.1371/journal.pgph.0002923 (PMC10901300; doi:10.1371/journal.pgph.0002923)
Supplement: S2 Table — (DOCX) [file pgph.0002923.s004.docx]

S2 Table: Composite variables used for constructing perceived safety practices (response variables), knowledge of safety practices, and risk scores (predictors).

| **Perceived relevance of safety practices scores (PRSP)** | **PPEs use** | **Knowledge of risk** | **Knowledge of safety** |
| --- | --- | --- | --- |
| - Checking their electrical connections before, during and after work - Protecting themselves and others from welding fumes - Protecting themselves and others from welding flash - Using safety goggles or welding screens during welding | - Use of Gloves - Use of safety goggles - Use of masks - Use of safety boots | - Knowledge of the kinds of metals that are used in the welding - Knowledge of any hazard due to welding cables and consumables kept in passageways - Knowledge about whether welding fumes cause any health problems | - Significance of proper earthing clamps and cable connections - Avoiding the causes of fire outbreaks at welding sites - Where to carry out welding activities safely - Not coiling or looping welding electrode cable around their neck/body - Removing electrodes from holders when not in use - Where water is not present - Not welding with wet hands |
