# Supplementary material for: Informal welders’ occupational safety and environmental health risks in northwestern Tanzania
Source: PLOS Glob Public Health. 2024 Feb 28;4(2):e0002923. doi: 10.1371/journal.pgph.0002923 (PMC10901300; doi:10.1371/journal.pgph.0002923)
Supplement: S3 Table — (DOCX) [file pgph.0002923.s005.docx]

**S3 Table:** Checklist on adherence to OHS Guidelines requirements as per OHS ACT 5 OF 2003; and its regulations (*tick or fill where applicable*)

| **S/N** | **Provision** | **Question** | **Response** | **Remarks** |
| --- | --- | --- | --- | --- |
| CI | Removal of dust and fumes (Section 61) | Are practicable measures are taken to protect the person employed against inhalation of fumes? | 1. Yes  2. No |  |
| C2 | Protection of eyes in welding (section 63 and fifth schedule) | Are goggles or welding screens while welding?  If no what other means do, they use? Mention………………………………  Is there a protective screen to protect others who are working in the same environment from welding flash? | 1. Yes  2. No  1. Yes  2. No |  |
| C3 | First aid Facilities | Is there a first aid box at the welding sites? | 1. Yes  2. No |  |
| C4 | Use of personal protective equipment’s | Are there any protective gears used in the welding site?  If Yes, list them ………………………………… | 1. Yes  2. No |  |
| C5 | Washing facilities | Is there any washing facility at the workplace? | 1. Yes  2.No |  |
| C6 | Sanitary conveniences | Are there any sanitary conveniences around/near the working area? | 1. Yes  2. No |  |

***Checklist Adopted from OSHA Tanzania (OSHA 2) Inspection Form***
