# Supplementary material for: Informal welders’ occupational safety and environmental health risks in northwestern Tanzania
Source: PLOS Glob Public Health. 2024 Feb 28;4(2):e0002923. doi: 10.1371/journal.pgph.0002923 (PMC10901300; doi:10.1371/journal.pgph.0002923)
Supplement: Attachment — Submitted filename: Responses to Reviewers_05.01.2024.docx [file pgph.0002923.s006.docx]

Journal Requirements:

1. Please include a complete copy of PLOS’ questionnaire on inclusivity in global research in your revised manuscript. Our policy for research in this area aims to improve transparency in the reporting of research performed outside of researchers’ own country or community. The policy applies to researchers who have travelled to a different country to conduct research, research with Indigenous populations or their lands, and research on cultural artefacts. The questionnaire can also be requested at the journal’s discretion for any other submissions, even if these conditions are not met.  Please find more information on the policy and a link to download a blank copy of the questionnaire here: https://journals.plos.org/globalpublichealth/s/best-practices-in-research-reporting. Please upload a completed version of your questionnaire as Supporting Information when you resubmit your manuscript.

Reviewer #1:

1. The introduction is very nice written but does not tell us why particulate matter and noise are factors chosen for this study. These factors are interesting, but not the most important issues among welders. Welders are well known to have problems with running eyes due to lack of eye protection, and also to have burns/injuries. Focusing on noise and particles does not seem logical.
   The aim of the study is unclear, as it is mentioned to see if the use of PPE improves health outcomes and reduces injury on job sites. There are no examinations of health and injuries in this manuscript. The aim should be revised.

**Response:**

It is very true that informal welders face many challenges, including running eyes, among others. The purpose of the paper is to understand how knowledge, experience, affect practice and by extension whether this reduces injuries.

Importantly, this is SELF-reported injury, not an examination.

We have detailed the short-term effects (such as cuts and burns, and irritation of the eyes, nose, chest, and respiratory tract) and long-term effects (such as the increased risk of pulmonary siderosis due to exposure to iron dust and cancer-causing agents found in welding smoke). This information is detailed on pages 4 – 5, lines 76 – 84.

2. PPE can be many types of, and the types needed for welders should have been described in the introduction.

**Response:**

We have added such a description on page 5, lines 88-94.

3. In methods, a questionnaire is mentioned briefly. It seems like the authors have not used any validated tool? The questionnaire needs to be described in detail if there is no reference the authors can give us that has these details described.

**Response:**

We have detailed the data collection tools and data collection sub-section on Page 8-9. We adopted a previous used questionnaire and checklists used by the Occupational and health and safety authorities, see line 176 – 190 where it reads, “A semi-structured questionnaire was adapted from a previous study that examined prevalence and determinants of occupational injuries among welders in small scale metal workshops in Wakiso District, Uganda (2021) and incorporating information from the *Tanzanian OSHA* *Fifth Schedule*, *sections 63,62,61, and 95 of OSH Act no 5 of 2003 and 2015 rules* (14,15) (See supplementary material 01). The survey also collected information on socio-demographic characteristics, including education level, history of working on welding sites, working hours, and age. Respondents answered a range of questions about: (1) knowledge of existing safety regulatory guidelines, (2) knowledge of occupational safety practices, (3) knowledge of occupational risks, and (4) perceived relevance of occupational safety practices (PRSP). Individual welders were asked about the area of the welding process and site where they worked for more than 90% of their time at the welding site.”

4. Composite scores are fine, but we need to know what the scores actually includes.

**Response:**

We have added a detailed explanation regarding the composition of the composite, see Page 9, line 191-198 where it reads, “Composite scores were created for occupational risk, (three yes/no questions), knowledge of occupational safety practices (10 questions, very important/important/not important), PRSP (four questions, relevant/not relevant). Specifically, there were three questions defining occupational risks, ten defining knowledge of occupational safety practices, and four defining PRSP (See supplementary material S2). Each question was binary coded (yes =1; no = 0) except for occupational safety practices, which had three responses (very important = 2; important =1 and not important = 0) and PRSP (relevant = 1; not relevant= 0).

5. Also, it is not described how the questionnaire was used. Did the workers answer them on paper, or were they interviewed? And by whom and where did this happen? Method details are missing here.

**Response:**

We have added such information, see page 8-9, line 176 – 190 where it reads, “A semi-structured questionnaire was adapted from a previous study that examined prevalence and determinants of occupational injuries among welders in small scale metal workshops in Wakiso District, Uganda (2021) and incorporating information from the *Tanzanian OSHA* *Fifth Schedule*, *sections 63,62,61, and 95 of OSH Act no 5 of 2003 and 2015 rules* (14,15) (See supplementary material 01). The survey also collected information on socio-demographic characteristics, including education level, history of working on welding sites, working hours, and age. Respondents answered a range of questions about: (1) knowledge of existing safety regulatory guidelines, (2) knowledge of occupational safety practices, (3) knowledge of occupational risks, and (4) perceived relevance of occupational safety practices (PRSP). Individual welders were asked about the area of the welding process and site where they worked for more than 90% of their time at the welding site.”

Also on page 10, line 205 – 208, see where it reads, “To enhance quality of the results, the survey was piloted to ten welders outside of the study area to inform the refinement of the survey and ensure questions were clear to the respondent. The questionnaire was administered orally in-person with responses recorded on paper during a face-to-face interview at the job site.”

7. A predefined checklist is also mentioned. It is such a good idea to use this method, but we need to know what the content of this was and how it was handled as a source for data.

**Response:**

Information regarding the checklist is now presented on page 10, line 205 – 220. See where it reads, “In addition, a pre-designed checklist was used by the data collector to capture information on adherence to OSH guidelines (See supplementary material 3). The inspection checklist, adapted from the *Tanzanian OSHA* *Fifth Schedule*, *sections 63,62,61, and 95 of OSH Act no 5 of 2003 and 2015 rules* (14,15), captured workers’ use of PPE (safety boots, safety goggle, mask, and gloves) during welding activities based on the type of activity and associated required PPE as specified by the American Safety and Health Fact Sheet for Welding and Cutting (American 2014). The checklist

also captured site hazards, including fire risks, lack of grounding, water on-site, sanitary conveniences, washing facilities, lack of first aid facilities, protection against dust and fumes and protection of eyes in welding (*see supplementary material S03*).”

8. Types of PPE are listed, but this is not sufficient. It is not always necessary with all types of PPE, this depends on the work done – therefore the authors need to describe how this observation was done. It is all very unclear.

**Response:**

We have revised to include such information, see page 10, line 208 – 224 where it reads, “a pre-designed checklist was used by the data collector to capture information on adherence to OSH guidelines (See supplementary material 3). The inspection checklist, adapted from the *Tanzanian OSHA* *Fifth Schedule*, *sections 63,62,61, and 95 of OSH Act no 5 of 2003 and 2015 rules* (14,15), captured workers’ use of PPE (safety boots, safety goggle, mask, and gloves) during welding activities based on the type of activity and associated required PPE as specified by the American Safety and Health Fact Sheet for Welding and Cutting (American 2014). The checklist also captured site hazards, including fire risks, lack of grounding, water on-site, sanitary conveniences, washing facilities, lack of first aid facilities, protection against dust and fumes and protection of eyes in welding (*see supplementary material S03*).Binary response for each PPE use: yes=1; no=0. The composite score for the PPE using the same equation as for the survey questions. The use of equipment was recorded as yes/no, as was the number of times a safety violation was observed.”

9. Air and noise pollution is shortly mentioned – but this description is not detailed enough, the reader cannot grasp the validity of this part of the study. Maybe the best idea for this study is to delete everything concerning these factors and concentrate on the interviews and observational study?

**Response:**

We have deleted this section as suggested. Thank you.

10. As for statistics, it is difficult to evaluate if the methods chosen are the best, since the data are insufficiently described.

**Response:**

We have detailed the data and data collection procedures. The tools are adopted from previous studies on similar population elsewhere among informal welders. See page 8 – 11.

11. The results table 1 shows that jobs performed were several? This should be explained. The figures are impossible to understand, e.g. there are 219 participants. 73 work with welding? And 100 work with all activities? What are all activities? And is this their daily work? Or the work they did on the day of the interview? Lack of details on the wording if questions and why the questions were asked makes table 1 difficult to read.

**Response:**

A footnote has been added (See Table 1) we have also added a description on ones’ working section, “Individual welders were asked about the area of the welding process and site where they worked for more than 90% of their time at the welding site.”). See page 9, line 186 – 188.

12. At the end of 3.1, we can read that the participants had frequently reported illnesses? Where did this come from, which questions? And what is respiratory complications? This should be removed from the study, it is not information well obtained – at least it is not possible to grasp how it was asked about.

**Response:**

We have omitted illness where applicable as suggested.

13. Statistical models are great, but simple descriptions of the data are needed to understand the analyses. Which PPE did the welders use, and how many used what? To refer to scores is not sufficient for the reader to understand the situation on these workplaces. Simple descriptions should be done before the statistical regression analyses.

**Response:**

Explanation on the data and data collection have been expanded on page 8 – 11 of the revised manuscript. We have added information regarding the PPE types among welders as per the American Safety and Health Fact Sheet, see page 10, line 208-224.

14. Table 2 and 3 and all the plots are not useful.
The result pages should be changed from text to tables. It is very difficult to see the results written like this in a text. This is especially so because we do not understand where the different variables came from, if it is from the questions or the observations. This needs to be totally rewritten and much simplified, and it is suggested to delete all about the noise and particles.

**Response:**

The noise data and particulate matters information have been removed as suggested by both reviewers.

15. The discussion also needs to be revised if the previous text is changed.

**Response:**

As per the explanation above, we have retained the discussion with slight modification. See page 19 – 23.

16. The study limitations seem strange and must be wrong and belong to another study perhaps? The health issues have been unclear in the whole manuscript and is here very confusing. The limitations must include the weaknesses in the methods, it seems you used not validated questions for instance. Selection of worker must be discussed.

**Response:**

We have revised the limitations and deleted some of them to conform with the findings of this study. See page 22, line 472 – 785.

17. The conclusion must be aligned with the aims of the study; but these must be made clear at the start.

**Response:**

The conclusion have been revised to align with the aim and findings of the study see page 23, line 487-506.

Reviewer #2:

1. On the title, I suggest you say ‘Occupational’ not occupation (Line 15).

**Response:**

We revised the title as suggested.

On the Abstract, I suggest you clearly state your objectives for instance numbering them for easy flow (Line 19 to Line 22).
**Response:**

We have revised the abstract based on the suggestion given.

2. On the Introduction, I suggest you insert some sources of your assertion. You can make use of sites such as International Labour Organization etc (Line 51).

**Response:**

3. I suggest you cite sources, at least two to affirm your statement on Line 68 to Line 70. Cite sources from PLOS Global Health Journal or any other reputable institutions such as the World Health Organisation.

**Response:**

Citations have been added as suggested.

4. I suggest you revisit Line 180 to Line 190

**Response:**

This information regarding noise and Particulate matter data collection has been deleted as advised earlier.

5. I suggest you in your results, you include not just tables but bar graphs and pie charts

**Response:**

We have included Fig 1 for same as suggested.
